# Targeted next-generation sequencing of dedifferentiated chondrosarcoma in the skull base reveals combined *TP53* and *PTEN* mutations with increased proliferation index, an implication for pathogenesis

**DOI:** 10.18632/oncotarget.9618

**Published:** 2016-05-26

**Authors:** Lu Gao, Xiafei Hong, Xiaopeng Guo, Dengfeng Cao, Xiaohuan Gao, Thomas F. DeLaney, Xinqi Gong, Rongrong Chen, Jianjiao Ni, Yong Yao, Renzhi Wang, Xi Chen, Pangzehuan Tian, Bing Xing

**Affiliations:** ^1^ Department of Neurosurgery, Peking Union Medical College Hospital, Chinese Academy of Medical Sciences and Peking Union Medical College, Beijing, China; ^2^ Department of General Surgery, Peking Union Medical College Hospital, Chinese Academy of Medical Sciences and Peking Union Medical College, Beijing, China; ^3^ Department of Pathology and Immunology, Washington University School of Medicine, St Louis, Missouri, USA; ^4^ Binhai Genomics Institute, BGI-Tianjin, Tianjin, China; ^5^ Tianjin Translational Genomics Center, BGI-Tianjin, Tianjin, China; ^6^ Department of Radiation Oncology, Massachusetts General Hospital, Harvard Medical School, Boston, Massachusetts, USA; ^7^ Institute for Mathematical Sciences, Renmin University of China, Beijing, China; ^8^ Institute of Basic Medical Sciences and School of Basic Medicine, Peking Union Medical College, Chinese Academy of Medical Sciences and Peking Union Medical College, Beijing, China; ^9^ Department of Medical Oncology, Peking Union Medical College Hospital, Chinese Academy of Medical Sciences and Peking Union Medical College, Beijing, China; ^10^ Department of Cancer Research, Jingke Biotech, Guangzhou, China

**Keywords:** dedifferentiated, chondrosarcoma, TP53, PTEN, proliferation

## Abstract

Dedifferentiated chondrosarcoma (DDCS) is a rare disease with a dismal prognosis. DDCS consists of two morphologically distinct components: the cartilaginous and noncartilaginous components. Whether the two components originate from the same progenitor cells has been controversial. Recurrent DDCS commonly displays increased proliferation compared with the primary tumor. However, there is no conclusive explanation for this mechanism. In this paper, we present two DDCSs in the sellar region. Patient 1 exclusively exhibited a noncartilaginous component with a *TP53* frameshift mutation in the pathological specimens from the first surgery. The tumor recurred after radiation therapy with an exceedingly increased proliferation index. Targeted next-generation sequencing (NGS) revealed the presence of both a *TP53* mutation and a *PTEN* deletion in the cartilaginous and the noncartilaginous components of the recurrent tumor. Fluorescence in situ hybridization and immunostaining confirmed reduced DNA copy number and protein levels of the *PTEN* gene as a result of the *PTEN* deletion. Patient 2 exhibited both cartilaginous and noncartilaginous components in the surgical specimens. Targeted NGS of cells from both components showed neither *TP53* nor *PTEN* mutations, making Patient 2 a naïve *TP53* and *PTEN* control for comparison. In conclusion, additional *PTEN* loss in the background of the *TP53* mutation could be the cause of increased proliferation capacity in the recurrent tumor.

## INTRODUCTION

Dedifferentiated chondrosarcoma (DDCS) is an uncommon type of chondrosarcoma with a peak incidence at 60 to 70 years of age [1]. The most frequently affected locations include the femur, pelvis, humerus and scapula. Skull involvement is extremely rare [1]. Morphologically, there are two distinct components. The cartilaginous component is characterized by well-differentiated chondrocyte-like tumor cells. In contrast, the noncartilaginous dedifferentiated component is characterized by spindle-shaped neoplastic cells [2]. DDCS can be further categorized into two subtypes based on the relationship between the two components. In the classical subtype [3, 4], the two components typically exhibit a sharp boundary with no transitional zone, whereas a clear transitional zone between the two components is observed in the non-classical type [4].

The histogenesis of DDCS remains unclear. More specifically, the origins of the cartilaginous and noncartilaginous components of DDCS remain unknown. Two theories exist with regard to the origins of the disease. The collision tumor theory [3, 5] states that each component originates separately from different progenitor cells. In contrast, the competing theory maintains that both components originate from the same progenitor cells and share common chondrosarcoma-associated somatic mutations [4, 6].

There is a dismal prognosis for this disease, for which surgical intervention remains the mainstay for treatment [1]. The value of chemotherapy remains questionable [7]. Previous studies indicate that DDCS might recur in the presence or absence of radiation therapy [2]. As previously reported [6, 8], the Ki-67 proliferation index increased when chondrosarcoma recurs; however, the mechanism remains unresolved. Previous studies [8, 9] have identified several frequent genetic mutations in DDCS, including *IDH1*, *IDH2*, and *TP53*. *TP53* gene deregulation has long been suggested as a causative factor for DDCS. The TP53 protein is frequently overexpressed in DDCS [4, 10, 11]. However, *TP53* alone cannot explain the increased Ki-67 index. Additional genetic or epigenetic events might account for the more rapid progression.

In this paper, we present two patients of DDCS in the skull region after radiation therapy. We used targeted next-generation sequencing (NGS) technology [12–15] to sequence a panel of genes in an attempt to discover targetable genetic changes and to decipher the pathogenesis of increased proliferation capacity in the recurrent tumor.

## RESULTS

### Medical history, radiographic findings, treatment and pathologic findings

#### Patient 1

**Figure 1:**
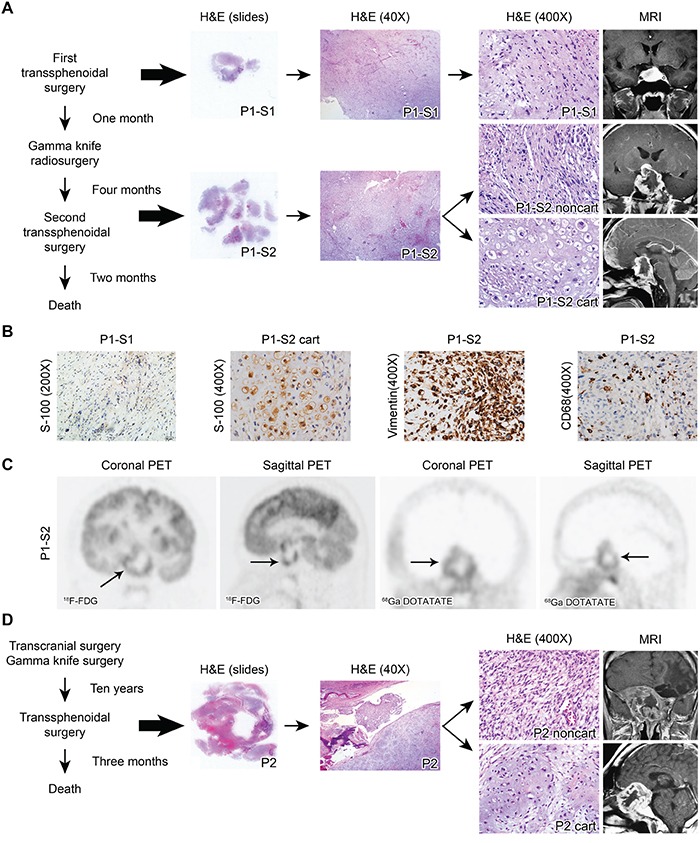
**A.** H&E staining and enhanced MRI of Patient 1. The surgical specimen from the first surgery showed only noncartilaginous spindle-shaped tumor cells (P1-S1, upper panel). The surgical specimen from the second surgery exhibited both cartilaginous (P1-S2 cart) and noncartilaginous (P1-S2 noncart) neoplastic components (lower panel). **B.** Representative immunostaining images for S-100 in P1-S1 as well as S-100, vimentin and CD68 in P1-S2. **C.** The FDG PET image demonstrated irregular ring-shaped tracer uptake in the sellar region (arrow, average SUV 2.58, SUVmax 5.16). Similarly, the DOTATATE PET image highlighted the same ring-shaped somatostatin receptor expression in the tumors (arrow, average SUV 0.67, SUVmax 1.04). **D.** H&E staining and MRI of Patient 2. The surgical specimen exhibited both cartilaginous (P2 cart) and noncartilaginous (P2 noncart) components.

This patient was a 28-year-old man who was admitted to a local hospital due to headache and diplopia. Magnetic resonance imaging (MRI) revealed a 2.8 × 1.9 × 1.8 cm-sized mass with homogeneous enhancement in the sellar region after gadolinium injection (Figure 1A). The patient underwent a trans-sphenoidal surgery at that hospital. Hematoxylin and eosin (H&E) staining of the resected tumor tissue (Patient-1-surgery-1 or P1-S1) revealed that the tumor cells had a spindle shape without any chondrocytic tumor cells. No tumor cells showed S-100 positivity by immunostaining (Figure 1B). No positivity was noted for neuron-specific enolase (NSE), glial fibrillary acidic protein (GFAP), epithelial membrane antigen (EMA), or actin in the tumor cells (data not shown). Therefore, no definitive diagnosis, except for a spindle cell tumor, was reached in the local hospital.

One month later, MRI detected tumor relapse, possibly derived from the remnant tumor cells. The patient then underwent a Gamma knife radiosurgery with a dose of 1260 cGy to the tumor region (50% isodose curve) at the local hospital in an attempt to control the recurrent tumor. Unfortunately, 4 months after the Gamma knife radiosurgery, the patient exhibited progressive decline in visual acuity, severe headaches and blepharoptosis. The patient was then transferred to our hospital, and MRI demonstrated a 3.0 × 4.8 × 3.5 cm irregular sellar cystic mass with ring-enhancement after gadolinium injection (Figure 1A). Both fluorodeoxyglucose (FDG) and tetraazacyclododecane tetraacetic acid–octreotate (DOTATATE) positron emission tomography (PET) images exhibited similar ring-shaped tracer uptake (Figure 1C). The patient then underwent a trans-sphenoidal surgery. The pathological specimen was defined as Patient-1-surgery-2 (P1-S2). H&E staining revealed both cartilaginous (P1-S2 cart) and noncartilaginous (P1-S2 noncart) components (Figure 1A). A clear transitional zone was noted between the two components, resembling the non-classical type DDCS. The cartilaginous cells exhibited S-100 positivity by immunohistochemical staining (Figure 1B), whereas the noncartilaginous component did not (data not shown). Both components exhibited vimentin positivity, indicating a mesenchymal origin (Figure 1B). CD68 staining was positive in a portion of the cells, most likely the intermixed histiocytes (Figure 1B). The P1-S2 noncartilaginous component exhibited a similar morphology to the tumor cells in P1-S1, indicating that P1-S2 might originate from the remnant tumor cells from P1-S1.

After surgery, although his vision improved, the patient died two months later due to disease progression.

#### Patient 2

This patient was a 37-year-old male patient admitted to a local hospital because of headache, coarsened facial features and enlarged hands and feet. He had a history of removal of a growth hormone-secreting pituitary adenoma by a transcranial approach followed by Gamma knife radiosurgery (exact dose of radiosurgery not documented) ten years previous. At the time of admission, the patient complained of severe cephalalgia and vision loss in the right eye. This patient was then admitted to our hospital. MRI revealed a 5.6 × 5.8 × 5.0 cm irregular cystic mass with ring-enhancement, extending to the sphenoid sinus, cavernous sinus, clivus, and suprasellar region (Figure 1D). The patient underwent a trans-sphenoidal surgery but died three months later. The postoperative pathologic diagnosis was DDCS consisting of both cartilaginous (P2 cart) and noncartilaginous (P2 noncart) components. A sharp boundary was noted with no transitional zone between the cartilaginous and noncartilaginous components, resembling the classical type (Figure 1D). The noncartilaginous spindled tumor cells were negative for MyoD1, myogenin, and desmin by immunohistochemical staining (data not shown), thus excluding the possibility that the noncartilaginous component was a variant of rhabdomyosarcoma [5].

### Sequencing and mutation detections

To address the question of whether the P1-S2 tumor arose from remnant tumor cells from P1-S1 or arose *de novo*, targeted NGS sequencing was conducted for the P1-S1 tissue, P1-S2 tissue and peripheral blood cell DNA. It should be noted that the P1-S2 tissue that was sequenced consisted of both cartilaginous and noncartilaginous components without further micro-dissection, as the cartilaginous component was interspersed among the noncartilaginous component and exhibited clear transitional zones in P1-S2.

**Figure 2:**
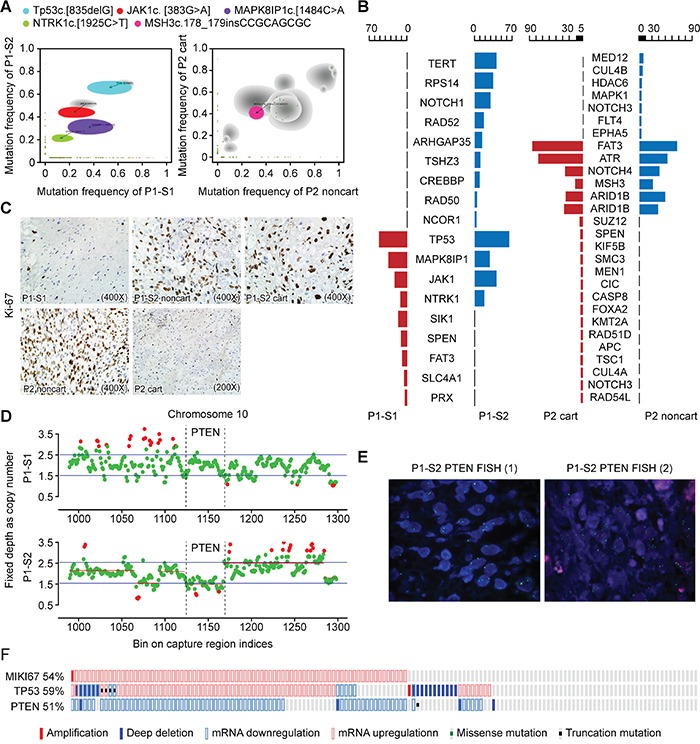
Targeted NGS showed a combined *TP53* and *PTEN* loss **A.** A comparison of the mutation allele frequency in the P1-S1 (x-axis) versus those in P1-S2 (y-axis) is shown (left panel). A comparison of the mutation allele frequency in the P2 noncart (x-axis) versus those in P2 cart (y-axis) is presented (right panel). Shaded areas represent Bayesian posterior probability distributions over mutation allele frequency in both samples from the same patients. Gray shading indicates mutation allele frequency distributions having considerable uncertainty, with lighter shading indicating greater uncertainty. Mutations of interest are also labeled. **B.** Oseq targeted sequencing revealed both shared and distinct mutations. **C.** Immunostaining of Ki-67 indexes in surgical specimens from P1-S1, P1-S2 noncart, P1-S2 cart, P2 noncart and P2 cart. **D.** Schematic view of copy number estimation from the targeted NGS depth data. The copy number of chr10 in the P1-S1 sample revealed no copy number changes in the *PTEN* gene region (locus: 1123~1166) (upper), whereas the copy number of chr10 in the P1-S2 sample showed alterations in the *PTEN* gene exon 1-9 region (lower). **E.** FISH revealed heterozygous and homozygous deletions in the *PTEN* gene in P1-S2, with organ probes detecting *PTEN* and green probes detecting the alpha satellite of 10p11.1-q11.1. **F.** Schematic views of the expression levels of MKI67, TP53 and PTEN in a sarcoma dataset.

Targeted NGS sequencing revealed shared frameshift mutations in *TP53* c.[835delG] and three other pathogenic missense mutations, *JAK1* c.[383G>A], *MAPK8IP1* c.[1484C>A] and *NTRK1* c.[1925C>T], in both P1-S1 and P1-S2, indicating that the tumor sample from the second surgery (recurrent tumor) was derived from the residual tissue from the first tumor. Notably, the mutation frequencies in *JAK1*, *NTRK1*, and *TP53* increased in P1-S2 compared with P1-S1 (Table 1; Figure 2A, left panel; Figure 2B, left panel). No detectable level of *IDH1/2* gene mutations was found in either P1-S1 or P1-S2, which is consistent with previous research demonstrating that not all chondrosarcomas harbor such mutations [9, 16].

**Table 1:** Sequencing data analyzed by the Standard BGI in-house NGS analysis for Patient 1

Gene name	cHGVS	pHGVS_ad	Mutation type	Mutation frequencies in P1-S1	Mutation frequencies in P1-S2	Condel	Pathogenicity
PRX	c.[1687G>A]	p.[A563T]	Missense	4.27	Not detectable	0.313	VUS
SLC4A1	c.[931C>A]	p.[L311I]	Missense	5.32	Not detectable	0.477	VUS
FAT3	c.[8657A>G]	p.[D2886G]	Missense	10	Not detectable	0.319	VUS
SPEN	c.[811G>A]	p.[G271S]	Missense	11.9	Not detectable	0.423	VUS
SIK1	c.[1355C>T]	p.[P452L]	Missense	16.67	Not detectable	0.523	Pathogenic
NTRK1	c.[1925C>T]	p.[A642V]	Missense	12.5	17.72	0.540	Pathogenic
JAK1	c.[383G>A]	p.[R128H]	Missense	23.53	41.48	N/A	Pathogenic^*^
MAPK8IP1	c.[1484C>A]	p.[A495D]	Missense	35.71	28.02	0.750	Pathogenic
TP53	c.[835delG]	p.[G279fs^*^?]	Frameshift	53.12	64.39	N/A	Pathogenic
NCOR1	c.[6591G>C]	p.[K2197N]	Missense	Not detectable	3.04	0.523	Pathogenic
RAD50	c.[323A>G]	p.[K108R]	Missense	Not detectable	4.06	0.396	VUS
CREBBP	c.[1974C>G]	p.[I658M]	Missense	Not detectable	8.87	0.592	Pathogenic
TSHZ3	c.[1804A>T]	p.[M602L]	Missense	Not detectable	10.36	0.395	VUS
ARHGAP35	c.[3779C>T]	p.[P1260L]	Missense	Not detectable	14.03	0.442	VUS
RAD52	c.[593C>T]	p.[P198L]	Missense	Not detectable	18.02	0.467	VUS
NOTCH1	c.[658G>A]	p.[V220M]	Missense	Not detectable	29.21	0.514	VUS
RPS14	c.[341C>T]	p.[S114L]	Missense	Not detectable	34.89	0.820	Pathogenic
TERT	c.[901C>T]	p.[R301C]	Missense	Not detectable	41.02	0.536	Pathogenic

Abbreviations: HGVS: Human Genome Variation Society; P1-S1: Patient-1-surgery-1; P1-S2: Patient-1-surgery-2; VUS: variants of unknown significance; N/A: Not available.

* SIFT=0.00, Polyphen-2=1.000 for JAK1: c.[383G>A]

Furthermore, both components in P1-S2 (60-80%) exhibited a considerably increased Ki-67 index compared with the non-cartilaginous component (less than 3%) in P1-S1 (Figure 2C), indicating that the tumor cells exhibited a higher degree of proliferation. P1-S2 also harbored other pathogenic mutations, such as missense mutations in *NCOR1, CREBBP, RPS14* and *TERT* genes, and a loss of copy number in *PTEN* exons 1-9, indicating that additional genetic alterations were acquired during disease progression (Figure 2D).

We next asked whether both components from Patient 2 harbored mutations in either *TP53* or *PTEN* genes. The targeted NGS revealed no pathogenic *TP53* or *PTEN* gene mutations in either P2 cart or P2 noncart. *NOTCH4* c.[3562G>A] was predicted to be pathogenic and present in both P2 cart and P2 noncart (Table 2; Figure 2A, right panel; Figure 2B, right panel). Because no peripheral blood DNA was available from this patient as a reference control, we could not determine whether the shared mutations were germline or somatic. For validation purposes, sequencing data from Patient 1 samples (P1-S1 and P1-S2) were also analyzed by MuTect v1.1.4 and Indel by Varscan v2.3.6. The mutations that were called were almost identical with those from the standard BGI in-house NGS analysis (Supplementary Table 2).

**Table 2:** Sequencing data analyzed by the Standard BGI in-house NGS analysis for Patient 2

Gene name	cHGVS	pHGVS_ad	Mutation type	Mutation frequencies in P2 cart	Mutation frequencies in P2 noncart	Condel	Pathogenicity
RAD54L	c.[460C>T]	p.[R154W]	Missense	3.03	Not detectable	0.749	Pathogenic
NOTCH3	c.[4448G>A]	p.[R1483H]	Missense	3.06	Not detectable	0.351	VUS
CUL4A	c.[1723C>T]	p.[H575Y]	Missense	3.16	Not detectable	0.657	Pathogenic
TSC1	c.[1039T>C]	p.[W347R]	Missense	3.24	Not detectable	0.591	Pathogenic
APC	c.[1678A>G]	p.[K560E]	Missense	3.27	Not detectable	0.593	Pathogenic
RAD51D	c.[61A>G]	p.[R21G]	Missense	3.29	Not detectable	0.450	VUS
KMT2A	c.[6415T>C]	p.[Y2139H]	Missense	3.38	Not detectable	0.543	Pathogenic
FOXA2	c.[69+1G>A]	N/A	Splice-5	3.42	Not detectable	N/A	VUS
CASP8	c.[319C>T]	p.[R107C]	Missense	3.45	Not detectable	0.452	VUS
CIC	c.[4634C>T]	p.[A1545V]	Missense	3.52	Not detectable	0.459	VUS
MEN1	c.[598G>A]	p.[G200S]	Missense	3.62	Not detectable	0.646	Pathogenic
SMC3	c.[1129G>A]	p.[A377T]	Missense	3.75	Not detectable	0.617	Pathogenic
KIF5B	c.[2822G>A]	p.[R941H]	Missense	4.07	Not detectable	0.649	Pathogenic
SPEN	c.[5996A>G]	p.[K1999R]	Missense	4.32	Not detectable	0.370	VUS
SUZ12	c.[1078C>T]	p.[R360C]	Missense	5.38	Not detectable	0.448	VUS
ARID1B	c.[2764A>T]	p.[M922L]	Missense	47.88	48.47	0.401	VUS
ARID1B	c.[1025C>T]	p.[A342V]	Missense	45.76	59.26	0.327	VUS
MSH3	c.[178_179 insCCGCAGCGC]	p.[63_64insAAP]	Cds-indel	31.82	40.58	N/A	VUS
NOTCH4	c.[3562G>A]	p.[D1188N]	Missense	46.77	50.37	0.574	Pathogenic
ATR	c.[982A>G]	p.[M328V]	Missense	87.27	62.56	0.463	VUS
FAT3	c.[10244T>A]	p.[L3415H]	Missense	96.48	77.2	0.321	VUS
EPHA5	c.[16C>A]	p.[P6T]	Missense	Not detectable	3.17	0.476	VUS
FLT4	c.[3106A>G]	p.[R1036G]	Missense	Not detectable	3.41	0.542	Pathogenic
NOTCH3	c.[458G>A]	p.[R153H]	Missense	Not detectable	3.52	0.530	Pathogenic
MAPK1	c.[76A>G]	p.[T26A]	Missense	Not detectable	3.57	0.492	VUS
HDAC6	c.[2764C>T]	p.[Q922^*^]	Nonsense	Not detectable	3.7	N/A	VUS^*^
CUL4B	c.[830A>G]	p.[E277G]	Missense	Not detectable	4.9	0.527	Pathogenic
MED12	c.[5746C>T]	p.[Q1916^*^]	Nonsense	Not detectable	6.25	N/A	Pathogenic

Abbreviations: HGVS: Human Genome Variation Society; P2 cart: Patient 2 cartilaginous component; P2 noncart: Patient 2 noncartilaginous component; VUS: variants of unknown significance; N/A: Not available.

* SIFT=0.25, Polyphen-2=0.557 for HDAC6: c.[2764C>T]

### Combined *TP53* and *PTEN* loss likely resulted in an increased proliferation index after radiotherapy

With regard to the pathogenicity, *TP53* c.[835delG] was reported in five carcinoma samples in the Catalogue of Somatic Mutations in Cancer database. The International Agency for Research on Cancer (IARC) database for *TP53* was investigated, and the codons close to this mutation were frequently mutated in various types of cancer [17].

The *PTEN* exon 1-9 region in Patient 1 revealed normal reads for P1-S1 but reduced reads for P1-S2 (Figure 2D), indicating a *PTEN* loss in P1-S2. To confirm this observation, fluorescence in situ hybridization (FISH) was performed, which showed both heterozygous and homozygous deletions in P1-S2, with 67% of cells exhibiting *PTEN* gene probe loss (11% homozygous deletions and 56% heterozygous deletions) in randomly selected fields (Figure 2E).

To test whether the *TP53* and *PTEN* alterations were related to cell proliferation, we searched for a differently tested sample on ‘cBioPortal’ (http://www.cbioportal.org/), which is an open-source, web-based analyzer [18, 19]. One dataset [20] consisting of over two hundred adult soft tissue sarcomas was analyzed, and the gene encoding the Ki-67 protein (*MKI67*) was elevated in 54% of the cases, all of which displayed a deregulation of either *PTEN* or *TP53* expression (Figure 2F).

**Figure 3:**
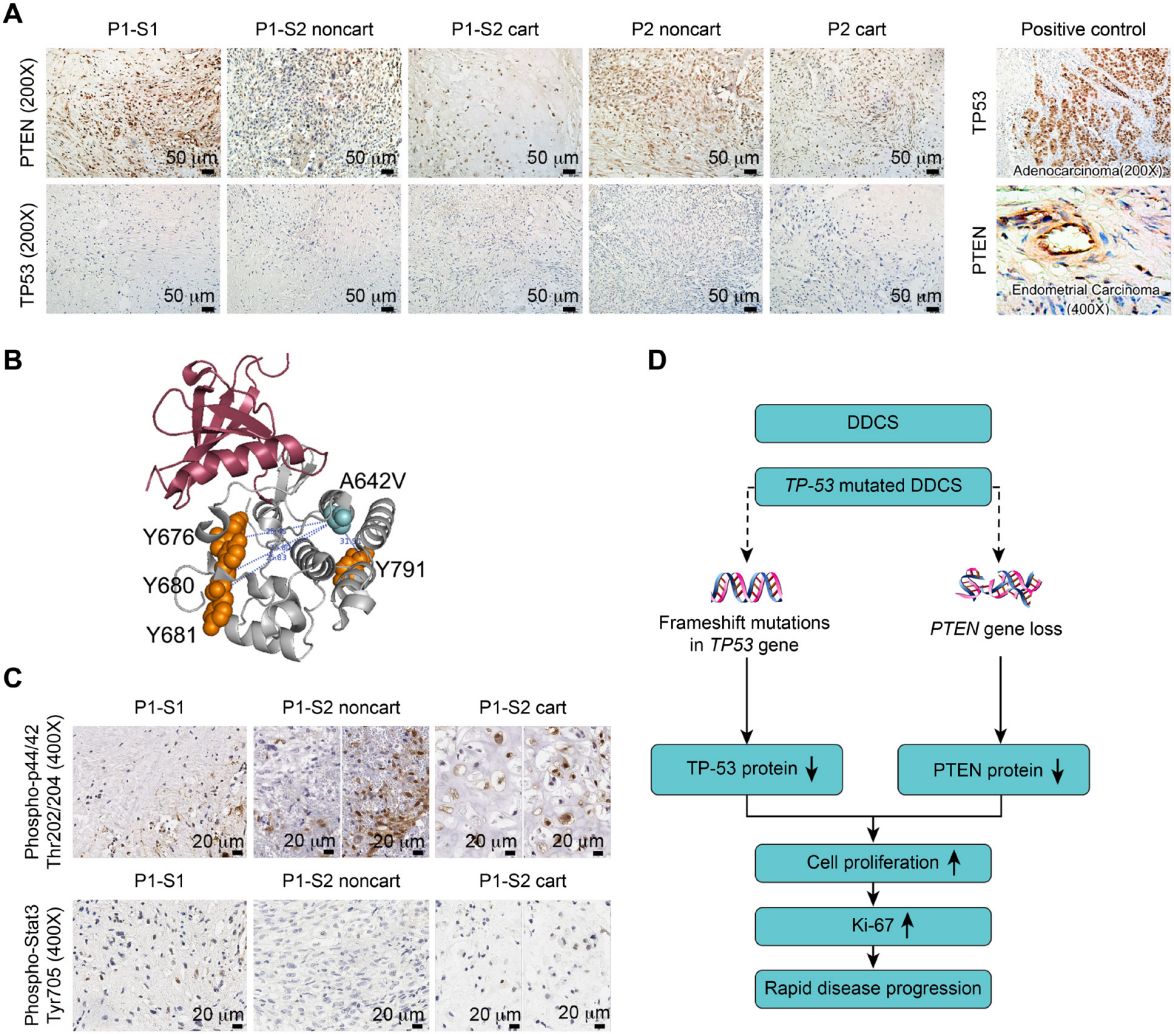
Combined TP53 and PTEN alterations accounted for disease progression **A.** Immunostaining of PTEN and TP53 in surgical specimens from P1-S1, P1-S2 noncartilaginous component (P1-S2 noncart), P1-S2 cartilaginous component (P1-S2 cart), Patient 2 noncartilaginous component (P2 noncart) and Patient 2 cartilaginous component (P2 cart) (left panel). Positive controls for TP53 and PTEN were adenocarcinoma and endometrial tumors, respectively (right panel). **B.** Three-dimensional (3D) structural view of the NTRK1 protein. The distance from mutated the A642V to the Y676, Y680, Y681, and Y791 residues were 25.48 Å, 23.80 Å, 25.83 Å, and 31.91 Å, respectively. **C.** Immunostaining of phospho-p44/42 (Thr202/204) and phospho-Stat3 (Tyr705). **D.** Proposed schematic illustration of the disease progression due to the combined *TP53* and *PTEN* changes.

Next, we examined the protein levels of PTEN and TP53 by immunostaining. Reduced PTEN protein levels were detected in immunostaining of P1-S2 (Figure 3A) [21]. Both P1-S1 and P1-S2 harbored the same frameshift mutation in *TP53*, which resulted in premature peptide termination and minimal immunostaining for the TP53 protein (Figure 3A). For Patient 2, neither the cartilaginous nor the noncartilaginous components exhibited *TP53* or *PTEN* mutations by sequencing. PTEN showed medium-strong immunostaining intensity in P2 cart and P2 noncart. TP53 revealed no immunostaining in either component in Patient 2 (Figure 3A). For quality control purposes, an adenocarcinoma sample was used as a positive control for TP53, and lymphocytes were used as a positive control for PTEN (Figure 3A).

### The other mutations in Patient 1 were not major contributors to disease progression

Because the *JAK1* and *NTRK1* mutation percentages were higher in P1-S2 compared with P1-S1, we were interested in whether these two mutations were accountable for the increased Ki-67 index. Recent literature has highlighted the targeting of receptor tyrosine kinases (RTKs) in human chondrosarcoma. One study [22] on human-derived chondrosarcoma cell lines revealed that targeted small molecule inhibitors for RTKs slow tumor cell growth. NTRK1 is a transmembrane protein receptor for nerve growth factor. After ligand binding, NTRK1 first auto-phosphorylates at five auto-phosphorylation residues (Y496, Y676, Y680, Y681, and Y791) and then phosphorylates downstream molecules, such as p44/42 [23, 24]. Activating mutations typically result from genomic rearrangements in papillary thyroid carcinoma and lung cancer [25], and missense mutations rarely lead to overactivation [23]. The *NTRK1* c.[1925C>T] resulted in a missense mutation of the NTRK1 protein p.[A642V] in the intracellular domain of the NTRK1 protein. We were able to visualize the spatial distances between the four auto-phosphorylated tyrosine residues and the amino acid change A642V (Figure 3B); the nearest distance of 23.80Å was between Y680 and A642V. Thus, the NTRK1 p.[A642V] mutation should not directly interfere with the phosphorylation process, and the phospho-p44/42 status should be independent of the NTRK1 p.[A642V] mutation. Interestingly, phospho-p44/42 exhibited positivity in approximately 45% of the cells of P1-S1. The staining pattern was not homogenous for this marker in P1-S2 noncartilaginous component; some areas exhibited more tumor cells that were positive for this marker than did others. The average percentage of tumor cells positive for this marker was 14%. Approximately 55% of the cells were positive in the P1-S2 cartilaginous component (Figure 3C). This result suggests that phospho-p44/42 might not be involved in the tumor progression. In addition, the *JAK1* c.[383G>A] mutation was previously identified in an endometrial carcinoma sample from the Cancer Genome Atlas (TCGA) project. Phospho-Stat3, a well-known target of the JAK1 protein, exhibited positivity in less than 5-8% of cells in the P1-S1, 3% of cells in the P1-S2 noncartilaginous component and 6% of cells in the P1-S2 cartilaginous component (Figure 3C). This result suggests that the *JAK1* mutation might not be an activating mutation in Patient 1.

We speculated that a *TP53*-mutated DDCS cancer cell might lose its surveillance over genomic instability after radiation, which might have induced a *PTEN* deletion in Patient 1. The combined *TP53* and *PTEN* changes further promoted tumor progression (Figure 3D).

## DISCUSSION

In this paper, we reported two patients of DDCS with mutation analysis of selected genes.

The main focus of the current study was to gain insight into the pathogenesis of disease progression of DDCS. The mechanism that contributes to the disease progression from the cartilaginous component to the non-cartilaginous sarcomatous component remains unclear. The present study proposed a mechanism for disease progression in which combined *TP53* and *PTEN* changes promote tumor progression in DDCS. Indeed, *PTEN* gene mutations are rare in chondrosarcoma, and Lin et al reported that a *PTEN* mutation was detected in only one out of forty chondrosarcoma cases [26]. Alterations in the *PTEN* gene are frequently observed after radiation when *TP53* is null or heterozygous in lymphoma [27]. *TP53* mutation or loss has been implicated in other types of sarcoma [28–30]. The concurrent loss of function in *PTEN* and *TP53* provided an explanation for rapid disease progression at the time of cancer recurrence for other tumors, which was also the case for invasive bladder cancer [31]. In addition, RTK has been reported to be activated in this type of tumor [22]. However, the *NTRK1* mutation proves not to be an activating mutation in Patient 1.

Both patients recurred after radiation therapy. Indeed, only a few cases of DDCS in the skull base have been reported, including two patients with a history of prior radiation therapy to the maxillary and frontal bone regions years before the onset of DDCS [9, 32]. The first issue is whether radiation ‘induced’ the occurrence of the dedifferentiated component in DDCS. Our study cannot answer this question with certainty. In general, conventional skull base chondrosarcomas respond well to surgery combined with radiation therapy [33, 34]. However, if the diagnosis is DDCS, then prognosis is poor Indeed, it is very challenging for a pathologist to make the diagnosis of DDCS when the cartilaginous component is absent or unavailable. Patient 1 exhibited nearly no morphological relevance to chondrosarcoma in the first surgery, possibly due to incomplete surgical resection, with only spindle-shaped tumor cells.

Previous studies have suggested that the noncartilaginous component might derive from the cartilaginous component with additional mutations. Ropke et al reported that LOHs of the *TP53* and *RB* genes were exclusively found in the noncartilaginous component [8]. Terek et al reported that likely-mutated TP53 protein was accumulated in the noncartilaginous but not cartilaginous components of DDCS [35]. Grote et al reported a *TP53* mutation that occurred exclusively in the noncartilaginous component [11]. Simms et al demonstrated increased TP53 staining in the spindle-cell portion of eight DDCS cases, whereas all of the cartilaginous components showed weak or no staining [36]. In our study, the non-cartilaginous component of P1-S1 shared common mutations in *TP53*, *JAK1*, *MAPK8IP1* and *NTRK1* in the mixed sample of P1-S2. However, the P1-S2 sample exhibited additional mutations in other genes, including a *PTEN* deletion. The TP53 immunostaining was negative for both Patient 1 and Patient 2. Patient 1 had a frameshift mutation in the *TP53* gene encoding pre-truncated TP53 proteins, which explains the negative TP53 immunostaining for this patient. For Patient 2, we speculate that the wild-type TP53 protein was low in abundance in comparison with the positive control and below the detection threshold of TP53 immunostaining in the current setting.

One major limitation of our study was that the number of sequenced genes was limited. The targeted NGS only included the predefined exonic regions of 508 genes. Therefore, epigenetic changes, genomic rearrangement, and genes outside of that panel could not be detected. Indeed, one study suggested that both genetic and epigenetic alterations might occur in DDCS [8].

In conclusion, the present study provided evidence that the rapid progression and increased Ki-67 index likely resulted from the concurrent mutations in *TP53* and *PTEN* genes. The multiple gene alternations detected in the present study suggest that there might be other molecular pathways contributing the progression of DDCS as well.

## MATERIALS AND METHODS

### Ethical approval

The study protocol was reviewed and approved by the Ethnical Committee of Peking Union Medical College Hospital. The two patients provided signed informed consent to approve the portion of the surgical specimens for research purposes.

### ^18^F-FDG and ^68^Ga DOTATATE PET/CT imaging

One patient was IV-injected with 370 MBq of ^18^F-FDG and 120 MBq of ^68^Ga DOTATATE (a novel somatostatin analog), respectively. One hour later, the PET/CT images were acquired using a Siemens Biograph TruePoint True V PET/CT (Knoxville, TN, USA) combining 64-slice CT with a supine position.

### Targeted NGS and validation

Tumor DNA was extracted from formalin-fixed paraffin-embedded (FFPE) samples and fresh frozen samples using the QIAamp DNA FFPE Tissue Kit and the DNEasy Blood and Tissue Extraction Kit (Qiagen, Hilden, Germany), respectively, according to the manufacturer's instructions. All FFPE and fresh frozen tissue samples underwent H&E staining and were reviewed by a pathologist to ensure > 70% tumor content. DNA purity and concentration were assessed using a NanoDrop2000 spectrophotometer (Thermo Fisher Scientific, Florida, USA); DNA quality was assessed by agarose gel electrophoresis.

Library construction was performed as previously described using 1 μg of DNA sheared by an ultrasonoscope to generate fragments with a peak length of 250 bps, followed by end repair, A tailing and ligation to the Illumina-indexed adapters according to the standard library construction protocol. Target enrichment was performed on a custom sequence capture-probe (Nimblegen, USA), which targeted 7,708 exons of 508 cancer-related genes and 78 introns from 19 genes recurrently rearranged in solid tumors, representing approximately 1.7 Mb of the human genome in total (Supplementary Table 1). Sequencing was performed with 2 × 101-bp paired-end reads and an 8-bp index read on an Illumina Hiseq 2500 platform (Illumina, San Diego, USA) using the manufacturer's protocols.

Primary sequence data were first processed by filtering adaptor sequences and removing low-quality reads using the SOAPnuke software developed by BGI and then aligned to build hg19 of the NCBI reference genome assembly using the BWA aligner v0.6.2-r126. Polymerase chain reaction (PCR) duplicate reads were removed by PICARD v1.98. Local realignment, base quality score recalibration was performed using GATK v2.3-9, and poorly mapped reads were removed based on the recalibration result. SNVs were detected by Standard BGI in-house NGS analysis SOMATK-SNV (developed by BGI, manuscript in preparation), and Indels (small insertions and deletions) were detected by SOMATK-INDEL (developed by BGI, manuscript in preparation). Copy number variation (CNV) calling was performed by CONTRA v2.0.4. Known SNPs and Indels obtained from the 1000 Genomes project were discarded. The remaining variants were subdivided into four categories: (1) variants that were previously reported to be deleterious; (2) variants that were likely to be gene-disrupting, including nonsense and splicing mutations and frameshift caused by Indels; (3) missense mutations that were judged to be pathogenic by Condel software [37] and those could not be predicted by Condel will be predicted by both SIFT and PolyPhen-2 [38, 39] (SIFT score < 0.05 AND Polyphen-2 score > 0.85); (4) the remaining variants that were considered as variants of unknown significance (VUS). The first three categories were defined as pathogenic. Sanger bidirectional sequencing confirmed the detected mutations of *NTRK1*, *JAK1*, *MAPK8IP1* and *TP53* in P1-S2. For comparison, SNVs were also called by MuTect v1.1.4 [40], and Indels were called by Varscan v2.3.6 [41].

### Pathological evaluation and immunohistochemistry staining

The FFPE specimens were serially sectioned to generate 4-μm unstained slides for H&E staining and immunohistochemical studies with the following markers: S-100 (Abcam, Cat #: ab34686, 1:250), vimentin (Abcam, Cat #: ab92547, 1:250), CD68 (Abcam, Cat #: ab955, 1:200), PTEN (Cell Signaling Technology, Cat #: 9559, 1:100), TP53 (Abcam, Cat #: ab4060, 1:100), Ki-67 (Cell Signaling Technology, Cat #: 9027 1:200), phospho-p44/42 (Thr202/Tyr204) (Cell Signaling, Cat #: 9101, 1:50), and phospho-Stat3 (Tyr705) (Cell Signaling Technology, Cat #: 9145, 1:400). The immunohistochemical staining was performed according to the standardized protocols [42, 43].

### FISH test for *PTEN* gene loss

The *PTEN* loss was confirmed by FISH (Vysis LSI PTEN SpectrumOrange/CEP 10 SpectrumGreen Probes Abbott, 07j74-001) with organ probes covering a 368-kb length of chromosome 10q23, including the regions encoding *PTEN*, and green probes covering CEP 10 of alpha satellite of 10p11.1-q11.1 DNA.

### Amino acid distance estimation from the protein 3D structure

The 3D protein structure file was downloaded from the RCSB Protein Data Bank. The distance was measured between the Ca atoms of the relative residues using PyMOL software.

## SUPPLEMENTARY TABLES




