# Supplementary material for: Targeted next-generation sequencing of dedifferentiated chondrosarcoma in the skull base reveals combined TP53 and PTEN mutations with increased proliferation index, an implication for pathogenesis
Source: Oncotarget. 2016 May 26;7(28):43557–69. doi: 10.18632/oncotarget.9618 (PMC5190044; doi:10.18632/oncotarget.9618)
Supplement: Supplementary file 1 [file oncotarget-07-43557-s001.pdf]

Targeted next-generation sequencing of dedifferentiated chondrosarcoma in the skull base reveals combined *TP53* and *PTEN* mutations with increased proliferation index, an implication for pathogenesis

SUPPLEMENTARY TABLES

Supplementary Table S1: Genes included in Oseq-T cancer panel

See Supplementary File 1

Supplementary Table S2: Patient 1: sequencing data analyzed by the MuTect and Varscan NGS analysis

Mutect/Varscan	P1-S1	chrom	coordinate	cHGVS	pHGVS	tumor_AF
SNV	NTRK1	chr1	156849033	c.1925C>T	p.A642V | p.Ala642Val	12%
	EXT2	chr11	44257853	c.2045G>A	p.R682Q | p.Arg682Gln	18.70%
	RPS14	chr5	149825219	c.341C>T	p.S114L | p.Ser114Leu	21.20%
	JAK1	chr1	65339153	c.383G>A	p.R128H | p.Arg128His	28.10%
	MAPK8IP1	chr11	45924982	c.1484C>A	p.A495D | p.Ala495Asp	40%
INDEL	TP53	chr17	7577100	c.835G[3>2] (std: c.835G[2] alt: c.837delG)	p.R280Efs*65 | p.Arg280Glufs*65	48.72%

Mutect/Varscan	P1-S2	chrom	coordinate	cHGVS	pHGVS	tumor_AF
SNV	RAD50	chr5	131911578	c.323A>G	p.K108R | p.Lys108Arg	3.10%
	CREBBP	chr16	3828151	c.1974C>G	p.I658M | p.Ile658Met	8.90%
	TSHZ3	chr19	31768895	c.1804A>T	p.M602L | p.Met602Leu	10.20%
	ARHGAP35	chr19	47440618	c.3779C>T	p.P1260L | p.Pro1260Leu	14%
	NTRK1	chr1	156849033	c.1925C>T	p.A642V | p.Ala642Val	18.30%
	RAD52	chr12	1025937	c.593C>T	p.P198L | p.Pro198Leu	18.80%
	MAPK8IP1	chr11	45924982	c.1484C>A	p.A495D | p.Ala495Asp	29.60%
	RPS14	chr5	149825219	c.341C>T	p.S114L | p.Ser114Leu	34.20%
	TERT	chr5	1294100	c.901C>T	p.R301C | p.Arg301Cys	41.30%
	JAK1	chr1	65339153	c.383G>A	p.R128H | p.Arg128His	42.40%
INDEL	TP53	chr17	7577100	c.835G[3>2] (std: c.835G[2] alt: c.837delG)	p.R280Efs*65 | p.Arg280Glufs*65	57.58%
